# Factors affecting decreased physical activity during the COVID-19 pandemic: an age-, gender-, and body mass index-matched study

**DOI:** 10.3389/fpubh.2023.1170049

**Published:** 2023-07-19

**Authors:** Jhin-Yi Shin, Jaemoo Lee, Jung-Min Lee, Nam Yoon Ho

**Affiliations:** ^1^Sports Science Research Center, Sungkyunkwan University, Suwon-si, Republic of Korea; ^2^Department of Physical Education, Kyung Hee University, Yongin-si, Republic of Korea; ^3^Sports Science Research Center, Kyung Hee University, Yongin-si, Republic of Korea; ^4^Teachers College, Jeju National University, Jeju, Republic of Korea

**Keywords:** community health survey, physical activity characteristic, COVID-19, public health, decreased physical activity

## Abstract

**Purpose:**

This study aims to investigate the association between factors affecting decreased physical activity (PA) during the COVID-19 pandemic by matching groups based on age, gender, and BMI variables using public Community Health Survey (CHS) data.

**Methods:**

Data from the CHS was selected and used to investigate health-related factors related to PA, including demographic, psychological, behavioral characteristics, sociocultural, and chronic disease. Exact group matching was conducted based on age, gender, and BMI variables. Frequency analysis, Chi-square test (*χ^2^* test), and multinominal logistic regression analysis were performed to analyze the data, and odds ratio (OR) and 95% confidence interval (95% CI) were presented. The study also examined the impact of COVID-19 on PA, the fear of PA infection.

**Results:**

The logistic regression analysis by gender showed that PA decreased in all age groups, males, and females during the COVID-19 pandemic. The decrease in PA was lower in age groups other than those aged 60 or older. Stress experience, residence area, housing type, drinking, smoking, education level, and fear of infection were found to affect decreased PA due to COVID-19. Specifically, experiencing stress (Odds Ratio [OR] = 1.178; 95% Confidence Interval [CI] = 1.054 ~ 1.317) and increased smoking (OR = 1.332; 95% CI = 1.073 ~ 1.653) had a slightly higher impact on decreased PA. Conversely, living in a suburban area (OR = 0.653; CI = 0.585 ~ 0.728), having public housing (OR = 0.836; CI = 0.754 ~ 0.928), having less than a high school education (OR = 0.813; CI = 0.729 ~ 0.907), staying the same with alcohol (OR = 0.567; CI = 0.507 ~ 0.633) and smoking (OR = 0.836; CI = 0.728 ~ 0.959), and having low fear of infection (OR = 0.817; CI = 0.737 ~ 0.905) had a slightly lower impact on decreased PA.

**Conclusion:**

PA should be maintained or increased, particularly in the context of social distancing measures during the pandemic. To ensure that PA can be sustained, a program should be developed that considers the individual’s geographical location, economic status, lifestyle, and environment.

## 1. Introduction

Regular physical activity (PA) has been linked to improved mental health (i.e., depression and anxiety) and a lower prevalence of chronic diseases and cancers (1). Reports suggest that the simple medical cost reduction from PA has an annual economic impact of 2 trillion won or more (2). Conversely, physical inactivity increases the risk of developing various diseases, resulting in higher medical costs (3). Furthermore, the value of PA was highlighted in the 2010 global mortality risk factor contribution survey, which revealed that inactivity was the leading cause of mortality (4).

On March 11, 2020, the World Health Organization (WHO) declared COVID-19 a global pandemic (5). This led to high-intensity social isolation, directly and indirectly affecting PA. As a precaution to stop the spread of the coronavirus, gyms, and sporting venues were closed nationwide (5). This reduced the opportunities for PA and increased the amount of time spent engaging in sedentary behavior (SB). Additionally, psychological fear due to the spread of infection had a negative impact on PA participation (6). Studies have shown that PA has decreased in several nations around the globe (7). According to the analysis of the Community Health Survey by the Korean Centers for Disease Control and Prevention, the rate of moderate or vigorous PA decreased by 4.9% in 2020 (19.8%) from 2019 (24.7%), and the rate of walking practice also decreased by 3.0% from 2019 (37.4%) to 2020 (40.4%). Furthermore, a study on the cessation of adult men’s and women’s PA found that 48.7% of men and 47.0% of women reported that they had stopped PA after COVID-19 (8).

Lack of PA is a well-known variable that contributes to obesity. A study on health determinants found that PA was the fourth most influential factor among other health behavior factors, and the most influential factor when combined with lack of PA and overweight and obesity (9). The population-attributable risk of PA to obesity-related chronic diseases, such as coronary artery disease, is estimated to be 39.5% (10). A 10% reduction in the lack of PA has been shown to reduce medical and economic losses (10). In response to this, the Korean government has implemented policies to promote PA since 2002 to prevent obesity and manage health.

Research examining the impact of COVID-19 on PA behavior is increasing. Stockwell et al. (11) conducted a systematic review of 66 cohort studies published until June 2020, which reported that the majority (64 out of 66) of studies indicated decreases in PA and increases in sedentary behavior (SB). Lesser and Nienhuis (12) emphasized the promotion of reduced PA by examining the impact of COVID-19 on Canadians’ PA behavior and well-being. Antunes et al. (13) explored lifestyle habits, PA, anxiety, and basic psychological needs in a sample of Portuguese adults during COVID-19, while Meyer et al. (14) studied changes in PA and sedentary behavior and mental health due to COVID-19 in US adults. Kim and Kang (15) conducted a literature review to analyze the decrease in PA due to COVID-19. Duncan et al. (16) examined health-related variables to a reduction in PA and its effects on mental health. Rossi et al. (17) conducted a systematic review of the evidence of the effects of the COVID-19 pandemic on children’s PA and their determinants and concluded that most studies indicated a decrease in PA. However, Rossi’s study did not include variables related to COVID-19, such as actual COVID-19 infection and COVID-19-related concerns. Togni et al. (18) found that household income was the only significant variable associated with the decline in PA during the COVID-19 pandemic, however, this study was limited due to its small sample size and bias toward Brazilians with high socioeconomic status. The 2020 Community Health Survey (CHS) is based on the PA-related variables categorized and presented by Sallis and Owen (19), Trost et al. (20) and Choi et al. (21).

This study examines the associations between PA reduction and the health-related variable by utilizing a matched-group design and data from the Korea Community Health Survey (KCHS). In the context of the COVID-19 pandemic, this research seeks to explore the relationship between factors and the decline in PA resulting from changes in leisure activities. By understanding these variables better, it is possible to strengthen the foundation for prevention or intervention.

## 2. Methods

### 2.1. Research participants

The 2020 Community Health Survey (CHS; General Statistics Approval No. 117075) conducted by the Korean Centers for Disease Control and Prevention was utilized for this study. This survey is based on Articles 4 and 2 of the Regional Health Act, which has been implemented as regional health statistics to support the development of public health plans since the implementation of the local government system in 1995. The survey is representative of adults aged 19 and older in Korea and was conducted using a systematic methodology. A total of 2,000 indicators, including health outcomes, health care system, health behavior, demographic and social characteristics, and socio-physical environment, were investigated annually through one-on-one interviews conducted by investigators trained at public health centers across the country. In 2020, COVID-19-related questions were added to the survey due to the epidemic of infectious diseases. Of the total of 229,269 adults aged 19 and older who participated in the CHS survey in 2020, the data of 207,122 individuals, excluding the data of 22,147 missing values for all variables used in the study, were first summarized. A random number table was generated from the data that was first organized, and analysis data were extracted through exact group matching based on gender, age, and BMI (Body Mass Index) variables to minimize the characteristic differences between the groups. A total of 31,360 participants were extracted (i.e., decreased PA; 15,680, non-decreased PA; 15,680).

### 2.2. Variables

In this study, the variable of decreased PA due to COVID-19 utilized a question directly asked in the CHS. According to the CHS guideline, The PA decrease questions were categorized as the “decreased group” if the response was “decreased” and the “non-decreased group” if the response was “similar” or “increased.” Additionally, six variables (demographic, psychological, behavioral characteristics, sociocultural, chronic disease, and corona-related variables) were selected as variables associated with the decrease in PA due to COVID-19. The demographic variable was categorized as male and female, and age was categorized as the 20s (19 ~ 29), 30s (30 ~ 39), 40s (40 ~ 49), 50s (50 ~ 59), and 60 years of age or older (60 or older). The mental health variable was divided into the ‘healthy group’ (i.e., very good, good) and the ‘unhealthy group’ (i.e., average, bad, very bad) for subjective health, and the ‘experienced group’ (i.e., very much experience, a great deal of experience) and the ‘inexperienced group’ (i.e., a small amount of experience, a small amount of experience) for subjective stress levels. The behavioral trait variable was categorized as ‘increased’, ‘stayed the same’, and ‘decreased’ for changes in drinking and smoking due to COVID-19. The sociocultural variable was categorized as ‘suburban areas’ and ‘urban areas’ for residential areas, ‘general housing’ and ‘apartments’ for housing type, and ‘less than high school graduate’ and ‘college graduate and above’ for education level. The chronic disease variable was categorized as the presence or absence of hypertension experience, diabetes experience, and obesity. Obesity was diagnosed using self-reported BMI factors and according to the obesity treatment criteria of the Korean Society of Obesity (22), with BMI less than 18.5 classified as ‘underweight’, 18.5 ~ 22.9 classified as ‘normal’, 23.0 ~ 24.9 classified as ‘overweight’, and 25.0 or higher classified as ‘obesity’. Subjects were then reclassified into ‘underweight + normal’, ‘overweight’, and ‘obesity’ groups. The COVID-19-related variable was categorized as ‘infected’ or ‘not infected’ for the presence or absence of COVID-19 infection, and ‘low fear (i.e., normal, not so, very not really that)’ or ‘high fear (i.e., very yes, yes)’ for fear of COVID-19 infection.

### 2.3. Data analysis

This study requested raw data from the Korean Centers for Disease Control and Prevention on August 8, 2022, and downloaded the data as a SAS file. The data was then transformed into SPSS Statistics 26.0 (SPSS Inc., Chicago, IL, USA) for analysis. A random number table was established in the 2020 CHS data, and exact group matching was performed based on gender, age, and BMI to minimize the characteristic differences. Frequency analysis was used to determine the demographic characteristics, and a chi-square test (χ^2^) was used to determine the difference in PA-related variables because of the reduction in PA caused by COVID-19. A multinomial logistic regression analysis was performed to examine the variables associated with the decrease in PA caused by COVID-19 Pandemic, and the odds ratio (OR) and 95% confidence interval (CI) were given. Statistical significance was set at *α* = 0.05.

## 3. Results

### 3.1. Participants characteristic information

This study included 31,360 adults aged 19 years and older, with an average age of 24.11 years in their 20s, 35.06 years in their 30s, 44.80 years in their 40s, 54.76 years in their 50s, and 70.12 years in their 60s and older. Table 1 presents a frequency analysis of location area, housing type, and education level for a total of 31,360 participants (i.e., decreased PA; 15,680, non-decreased PA; 15,680) as well as a descriptive statistical analysis for participants’ anthropometric information. In the suburban area, 42.0% of participants were in the decreased PA group, while 58.0% were in the non-decreased PA group. In the urban area, 54.6% of participants were in the decreased PA group and 45.5% were in the non-reduced PA group. In terms of housing type, 46.4% of participants were in the decreased PA group and 53.6% were in the non-decreased PA group in the general housing population. In apartments, 53.9% of participants were in the decreased PA group and 46.1% were in the non-decreased PA group. In terms of education level, 47.5% of participants were in the decreased PA group and 52.5% were in the non-decreased PA group in the Below high school, and in college and beyond, 52.4% of participants were in the decreased PA group and 47.6% were in the non-decreased PA group. Gender, age, and BMI were matched between groups (i.e., decreased PA and non-decreased PA), and there was no statistically significant difference in participants’ demographic information between groups (*p <* 0.05).

**Table 1:** Characteristics of participants in two groups.

Variables		Decreased PA (*n* = 15,680)		Non-decreased PA (*n* = 15,680)		Total (*n* = 31,360)	
		*n* (%)	M ± SD	*n* (%)	M ± SD	*n* (%)	M ± SD
Age (years)	Male		45.56 ± 16.07		45.72 ± 16.44		45.67 ± 16.23
	Female		45.72 ± 16.38		46.00 ± 16.82		45.86 ± 16.63
	Total		45.64 ± 16.26		45.89 ± 16.60		45.76 ± 16.43
BMI (kg/m^2^)	Male		25.10 ± 3.34		25.02 ± 3.43		25.06 ± 3.38
	Female		24.49 ± 3.82		24.49 ± 3.96		24.49 ± 3.89
	Total		24.80 ± 3.60		24.49 ± 3.89		24.78 ± 3.66
Location							
	Suburban	4,820 (42.0)		6,665 (58.0)		11,485 (36.6)	
	Urban	10,860 (54.6)		9,015 (45.4)		19,875 (63.4)	
Housing type							
	General housing	7,486 (46.4)		8,664 (53.6)		16,150 (51.5)	
	Apartment	8,194 (53.9)		7,016 (46.1)		15,210 (48.5)	
Education							
	High school and less	7,260 (47.5)		8,039 (52.5)		15,299 (48.8)	
	College and above	8,420 (52.4)		7,641 (47.6)		16,061 (51.2)	

The total number of study participants was 31,360, and the frequency analysis and descriptive statistics analysis were conducted to review the demographic characteristics according to decreased and non-decreased in PA due to COVID-19. The values of region, housing type and education were expressed as frequency (*n*) and percentage (%), and the values of age and body composition were presented as the mean ± standard deviation.

### 3.2. Decrease in PA due to COVID-19 and differences in variables related to PA

Table 2 demonstrates a decrease in PA due to COVID-19 and differences in variables related to PA. In the relationship between non-decreased PA and subjective health, males in the healthy group had the highest rate (48.2%) and the unhealthy group had the lowest rate (28.4%). Females in the unhealthy group experienced the greatest decrease in PA (26.7%), whereas the healthy group experienced the least decrease (23.3%). In the relationship between decreased PA and stress between males and females, the stress-inexperienced group had the highest non-decreased PA (males: 38.9%, females: 37.9%) and the stress-experienced group had the lowest non-decreased PA (males: 11.1%, females: 12.1%). The decrease in PA due to COVID-19 was high in both males and females with decreased alcohol consumption (male: 25.8%, female: 24.0%) and stay the same smoking group (male: 33.5%, female: 25.6%). Additionally, Urban area (males: 34.5%, females: 34.8%) and apartment (males: 26.1%, females: 26.1%) had a high decrease rate of PA. Lower middle school students had the lowest decrease in PA (males: 6.1%, females: 10.1%), while university graduates and over had the highest decrease in PA (males: 28.9%, females: 24.8%). Diabetes and hypertension did not demonstrate significant validation. Significant evidence of obesity and a decline in PA because of COVID-19 was found in both males (*χ*^2^ = 7.968, *p* < 0.05) and females (*χ*^2^ = 7.968, *p* < 0.05) respectively. Compared to the underweight + normal group, the obese group experienced decreased PA more frequently. In the no group, COVID-19 infection was diagnosed more frequently than in the yes group (the investigation period was early in the COVID-19 era). Significant levels of COVID-19 infection fear and a decline in PA as a result of COVID-19 were found in both males (*χ*^2^ = 33.259, *p* < 0.001) and females (*χ*^2^ = 62.249, *p* < 0.001). Males in the high fear group reported a decrease in PA of 32.4%, while females reported a decrease of 38.8%. Males in the low fear group reported a decrease in PA of 17.6 and 11.2%, respectively.

**Table 2:** The difference between gender × variables and decreased PA due to COVID-19.

Variables		PA group		Total	χ2	*p*
		Decreased *n* (%)	Non decreased *n* (%)			
Subjective health status						
Healthy group	Male	4,588 (41.9)	4,727 (43.2)	9,315 (85.1)	5.514	0.023
Unhealthy group		3,252 (29.7)	3,112 (28.4)	6,364 (58.1)		
Healthy group	Female	3,650 (23.3)	3,863 (24.6)	7,513 (47.9)	11.594	0.001
Unhealthy group		4,190 (26.7)	3,977 (25.4)	8,167 (52.1)		
Stress						
Experienced group	Male	2,009 (12.8)	1,741 (11.1)	3,750 (23.9)	25.174	0.001
Inexperienced group		5,831 (37.2)	6,099 (38.9)	11,930 (76.1)		
Experience group	Female	2,381 (15.2)	1,897 (12.1)	4,278 (27.3)	75.303	0.001
Inexperienced group		5,459 (34.8)	5,943 (37.9)	11,402 (72.7)		
Binge Alcohol consumption						
Increased	Male	525 (4.6)	278 (2.4)	803 (7.0)	347.775	0.001
Stayed the same		2,305 (20.0)	3,236 (28.1)	5,541 (48.2)		
Decreased		2,968 (25.8)	2,194 (19.1)	5,162 (44.9)		
Increased	Female	366 (4.6)	196 (2.5)	562 (7.1)	113.524	0.001
Stayed the same		1,793 (22.6)	2,130 (26.9)	3,923 (49.5)		
Decreased		1,903 (24.0)	1,542 (19.4)	3,445 (43.4)		
Smoking						
Increased	Male	422 (6.0)	232 (3.3)	654 (9.4)	110.293	0.001
Stayed the same		2,334 (33.5)	2,780 (39.8)	5,114 (73.3)		
Decreased		678 (9.7)	531 (7.6)	1,209 (17.3)		
Increased	Female	57 (6.8)	25 (3.0)	82 (9.7)	35.827	0.001
Stayed the same		216 (25.6)	337 (39.9)	553 (65.5)		
Decreased		115 (13.6)	94 (11.1)	209 (24.8)		
Location						
Suburban	Male	2,432 (15.5)	3,321 (21.2)	5,753 (36.7)	216.989	0.001
Urban		5,408 (34.5)	4,519 (28.8)	9,927 (63.3)		
Suburban	Female	2,388 (15.2)	3,344 (21.3)	5,732 (36.6)	251.316	0.001
Urban		5,452 (34.8)	4,496 (28.7)	9,948 (63.4)		
Housing type						
General housing	Male	3,746 (23.9)	4,374 (27.9)	8,120 (51.8)	100.737	0.001
Apartment		4,094 (26.1)	3,466 (22.1)	7,560 (48.2)		
General housing	Female	3,740 (23.9)	4,290 (27.4)	8,030 (51.2)	77.214	0.001
Apartment		4,100 (26.1)	3,550 (22.6)	7,650 (48.8)		
Education						
High school and less	Male	3,301 (21.0)	3,682 (23.5)	6,983 (44.5)	37.479	0.001
College and above		4,539 (28.9)	4,158 (26.5)	8,697 (55.5)		
High school and less	Female	3,959 (25.3)	4,357 (17.8)	8,316 (53.1)	40.559	0.001
College and above		3,881 (24.8)	3,483 (22.2)	7,364 (47.0)		
Hypertension						
Yes	Male	1,612 (10.3)	1,609 (10.3)	3,221 (20.5)	0.004	0.953
No		6,228 (39.7)	6,231 (39.7)	12,459 (79.5)		
Yes	Female	1,456 (9.3)	1,549 (9.9)	3,005 (19.2)	3.561	0.059
No		6,384 (40.7)	6,291 (40.1)	12,675 (80.8)		
Diabetes						
Yes	Male	732 (4.7)	708 (4.5)	1,440 (9.2)	0.440	0.507
No		7,108 (45.3)	7,132 (45.5)	14,240 (90.8)		
Yes	Female	601 (3.8)	654 (4.2)	1,255 (8.0)	2.433	0.119
No		7,239 (46.2)	7,186 (45.8)	14,425 (92.0)		
Obesity						
Underweight + Normal	Male	2,060 (13.1)	2,146 (13.7)	4,206 (26.8)	7.968	0.047
Overweight		1,860 (11.9)	1,774 (11.3)	3,634 (23.2)		
Obese		3,920 (25.0)	3,920 (25.0)	7,840 (50.0)		
Underweight + Normal	Female	3,009 (19.2)	2,963 (18.9)	5,972 (38.1)	8.978	0.030
Overweight		1,011 (6.4)	957 (6.1)	1,968 (12.6)		
Obese		3,920 (25.0)	3,920 (25.0.)	7,840 (50.0)		
COVID-19 infection diagnosis						
Yes	Male	76 (0.5)	36 (0.2)	112 (0.7)	14.388	0.001
No		7,764 (49.5)	7,804 (49.8)	15,568 (99.3)		
Yes	Female	49 (0.3)	39 (0.2)	88 (0.6)	1.143	0.285
No		7,791 (49.7)	7,801 (49.8)	15,592 (99.4)		
Concerns about COVID-19 infection						
Low fear	Male	2,756 (17.6)	3,105 (19.8)	5,861 (37.4)	33.259	0.001
High fear		5,084 (32.4)	4,734 (30.2)	9,818 (62.6)		
Low fear	Female	1,763 (11.2)	2,191 (14.0)	3,954 (25.2)	62.249	0.001
High fear		6,077 (38.8)	5,645 (36.0)	11,722 (74.8)		

The values are presented as the frequency (*n*) and percentage (%). Cross analysis were conducted to compare the differences between the variables of PA due to COVID-19. The derived value of *p* was found to be statistically significant (*p* < 0.001).

### 3.3. Effects of health-related variables with decreased PA due to COVID-19

**Figure 1:**
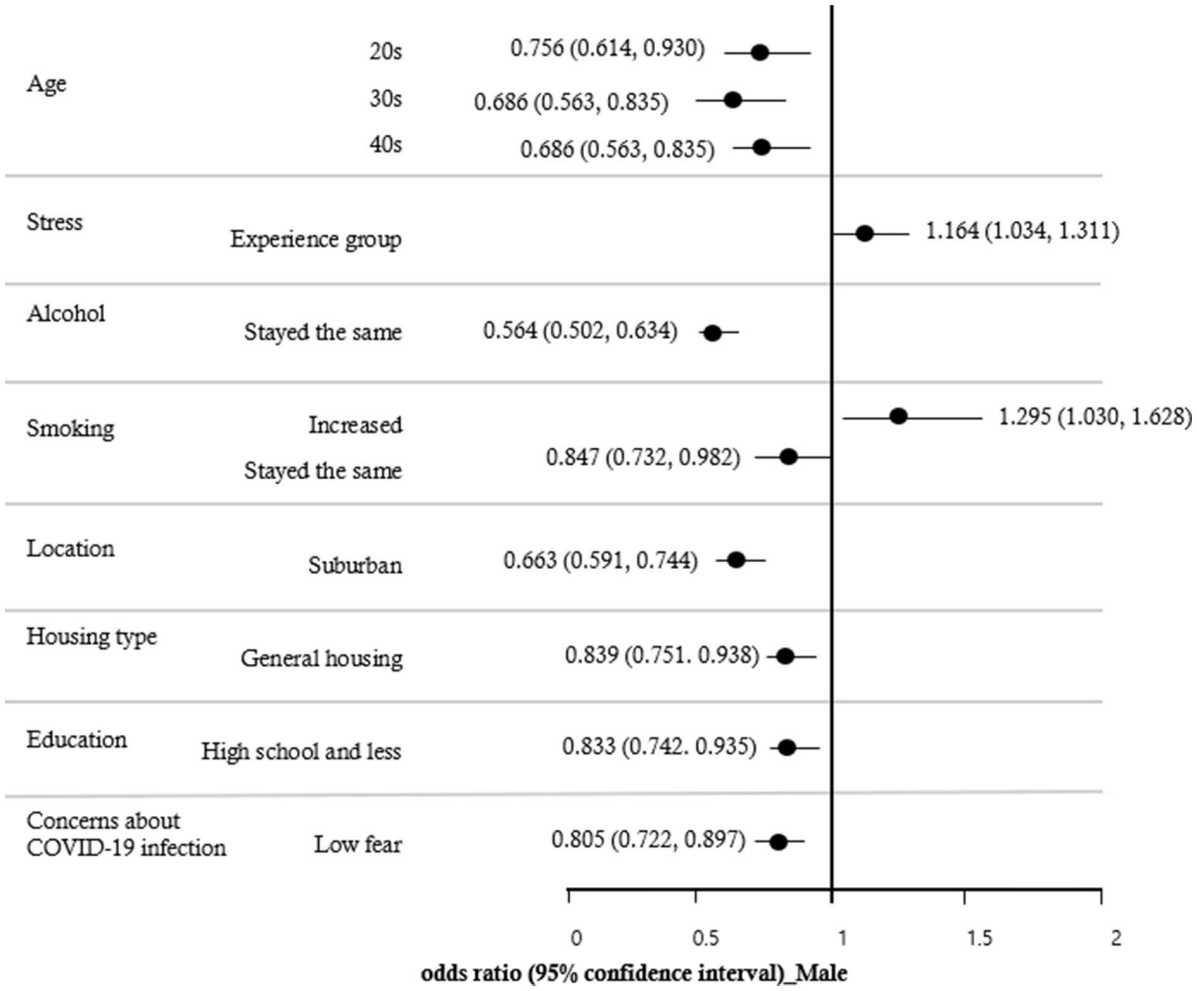
Forest plot of the odds ratios for decreased PA due to COVID-19 factors in male.

**Figure 2:**
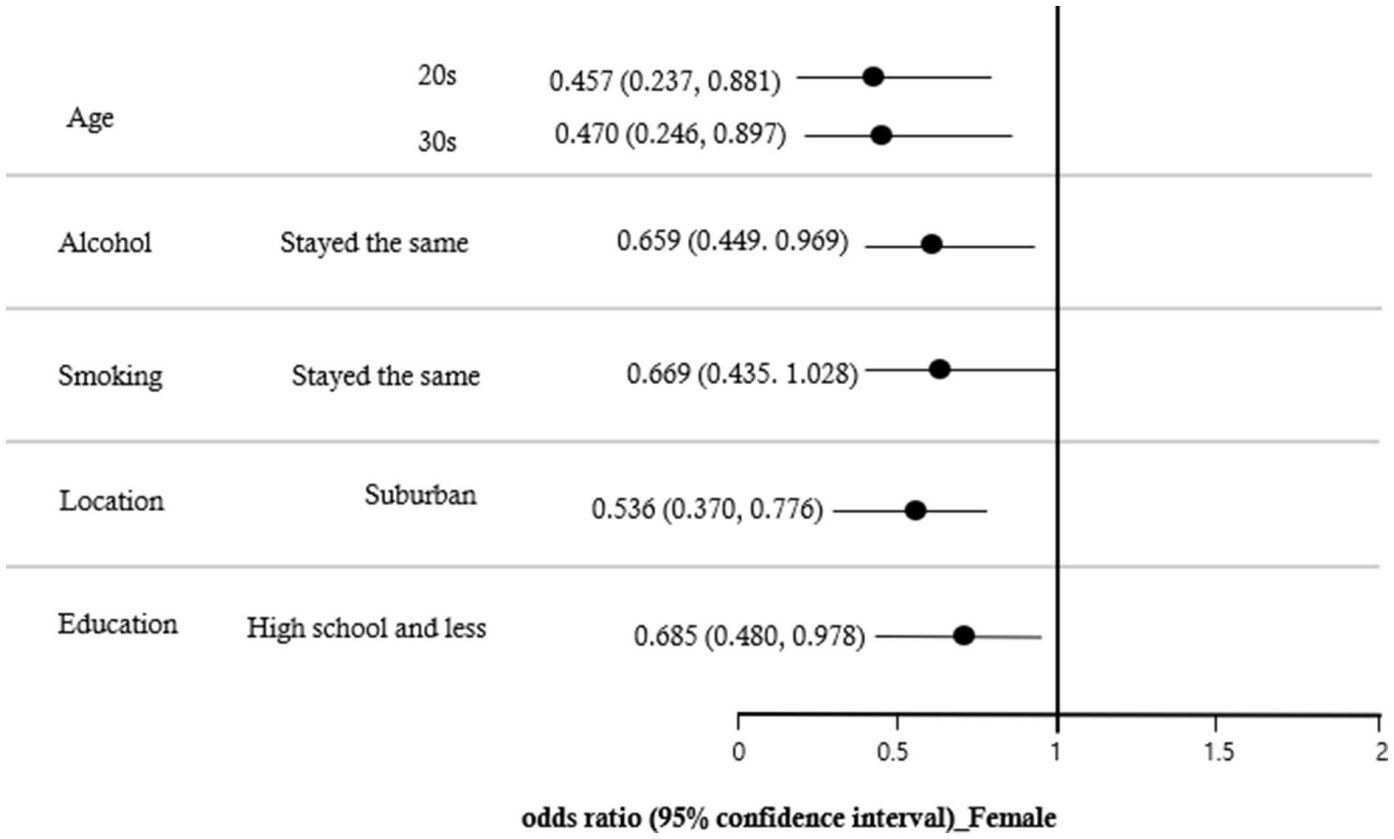
Forest plot of the odds ratios for decreased PA due to COVID-19 factors in female.

A multiple logistic regression analysis was conducted to identify the variables influencing the decrease in PA during the COVID-19 pandemic. The results, shown in Table 3, indicated that the effects of decreased PA due to COVID-19 were significantly different among those in their 20s (OR = 0.690; CI = 0.568 ~ 0.890), 30s (OR = 0.651; CI = 0.540 ~ 0.785), and 40s (OR = 0.745; CI = 0.624 ~ 0.890) compared to those in their 60s or older. The group that experienced stress (OR = 1.178; CI = 1.054 ~ 1.317) had a higher impact on decreased PA due to COVID-19 than the group that did not experience stress. The effect of alcohol on the decreased PA due to COVID-19 was lower when the alcohol consumption stayed the same (OR = 0.567; CI = 0.507 ~ 0.633) than when it decreased. The effects of smoking were lower when smoking stayed the same (OR = 0.836; CI = 0.728 ~ 0.959) than when smoking consumption decreased, and higher when it increased (OR = 1.332; CI = 1.073 ~ 1.653). The effect of location on decreased PA due to COVID-19 was lower in suburban areas (OR = 0.653; CI = 0.585 ~ 0.728) than in urban areas. The effect of housing type was lower in general housing (OR = 0.836; CI = 0.754 ~ 0.928) than in an apartment. In addition, the effect of decreased PA due to COVID-19 was lower in the group with less than a high school (OR = 0.813; CI = 0.729 ~ 0.907) than in the group with a college and graduate degree. In comparison to the group with high fear of COVID-19 infection, decreased PA was lower in the group with lower fear (OR = 0.817; CI = 0.737 ~ 0.905). There was no discernible impact from obesity, subjective health status, or the COVID-19 infection diagnosis. The results of examining the effects of study variables on the decreased PA due to COVID-19 by gender are shown in Figures 1, 2. The results for males in Figure 1 were similar to those for all participants in Table 3. As shown in Figure 2, the decreased PA among females due to COVID-19 was affected by age, location, alcohol, smoking, and education. Females in their 20s (OR = 0.457; CI = 0.237 ~ 0.881) and 30s (OR = 0.470; CI = 0.246 ~ 0.897) had lower odds ratios of reduced PA than those in their 60s or older. Additionally, suburban areas (OR = 0.536; CI = 0.370 ~ 0.776) had lower odds ratios than urban areas, and those with less than a high school (OR = 0.685; CI = 0.480 ~ 0.978) had lower odds ratios than college and graduate degrees.

**Table 3:** Odds ratios of decreased PA due to COVID-19 and predictors using multiple logistic regression (Total *n* = 31,360).

Variables	*B*	SE	*t*	*p*	OR	95% CI	
						Lower	Upper
Intercept	0.943	0.109	75.032	< 0.001			
Age							
20s	−0.371	0.099	14.027	< 0.001	0.690	0.568	0.838
30s	−0.429	0.095	20.303	< 0.001	0.651	0.540	0.785
40s	−0.294	0.090	10.584	0.001	0.745	0.624	0.890
50s	−0.132	0.090	2.133	0.144	0.876	0.734	1.046
60s≤	0^a^						
Obesity							
Underweight + Normal	0.088	0.060	2.177	0.140	1.092	0.971	1.228
Overweight	0.066	0.064	1.061	0.303	1.069	0.942	1.212
Obese	0^a^						
Subjective health status							
Healthy group	−0.032	0.052	0.369	0.543	0.969	0.874	1.073
Unhealthy group	0^a^						
Stress							
Experienced group	0.164	0.057	8.298	0.004	1.178	1.054	1.317
Inexperienced group	0^a^						
Alcohol consumption							
Increased	0.128	0.107	1.444	0.229	1.137	0.922	1.402
Stayed the same	−0.568	0.057	100.456	< 0.001	0.567	0.507	0.633
Decreased	0^a^						
Smoking							
Increased	0.287	0.110	6.769	0.009	1.332	1.073	1.653
Stayed the same	−0.180	0.070	6.529	0.011	0.836	0.728	0.959
Decreased	0^a^						
Location							
Suburban	−0.426	0.056	58.457	< 0.001	0.653	0.585	0.728
Urban	0^a^						
Housing type							
General housing	−0.179	0.053	11.297	0.001	0.836	0.754	0.928
Apartment	0^a^						
Education							
Less than high school	−0.206	0.056	13.689	< 0.001	0.813	0.729	0.907
College and Graduate degree	0^a^						
Concerns about COVID-19 infection							
Low fear	−0.203	0.053	14.856	< 0.001	0.817	0.737	0.905
High fear	0^a^						
COVID-19 infection diagnosis							
Yes	0.465	0.308	2.273	0.132	1.592	0.870	2.912
No	0^a^						

^a^Set to zero because this parameter is redundant. The reference category is “Non decreased PA.”

## 4. Discussion

This study aims to examine the associations between self-reported risk factors and decreased PA during the Pandemic. Data from the Community Health Survey (CHS) was utilized and analyzed based on matched age, gender, and body mass index (BMI) to minimize any potential differences in characteristics.

This study found that people aged 60 and over had a higher risk of decreased PA compared to those aged 20–40 due to the COVID-19 pandemic. Additionally, the findings revealed that the older adult had a higher rate of concern about coronavirus infection. These results support the idea that the older adult are more vulnerable to decreased PA and increased mortality due to COVID-19 infection (22). It has been suggested that the fear of infection has caused increased anxiety and decreased PA (23).

A statistically significant effect was not found. However, it was confirmed that decreased PA due to COVID-19 was higher in the underweight and normal groups than in the obese and overweight groups. It is hypothesized that those who had higher levels of PA before the pandemic experienced a greater decrease in PA due to activity restrictions. Multiple studies have supported this hypothesis, which has repeatedly shown a decrease in PA during restriction periods (24–27). Countries around the world are addressing PA as a result of COVID-19, and research is linking it to physical, psychological, and mental health (28–32).

Additionally, stress experience, residence area, housing type, drinking and smoking, education level, and fear of infection were found to affect decreased PA due to COVID-19. Specifically, experiencing stress, living in an urban area, living in an apartment, increasing alcohol and smoking, having a high level of education, and having a high fear of infection were found to have a greater effect on decreased PA.

The relationship between economic support for leisure activities and PA participation is well-documented (33). Higher levels of education and socioeconomic status are associated with an increased likelihood of engaging in PA through financial investment (33). However, due to the implementation of measures such as the cancellation of mass gatherings, closure of public spaces, stay-at-home orders (lockdown), and protective mask use in public spaces (34), sports facilities were closed and leisure activities were restricted, leading to a decrease in PA among those with higher levels of education (35). In contrast, those with lower levels of education had higher PA during work hours than leisure time, resulting in a smaller decrease in PA due to the coronavirus (36).

The place of residence may be a contributing factor to decreased PA during the Covid-19 pandemic. Beck et al. (37)found that suburban areas offer more suitable areas for sports activity within a limited radius and more space to practice PA. Additionally, the density of apartments in urban areas may increase the risk of infection spread, leading to higher concerns about COVID-19 (39). Psychological factors such as stress, depression, and anxiety (39) may also negatively impact PA motivation. Other variables, such as obesity (40, 41) and alcohol and smoking patterns (42), are known to have detrimental effects on participation in physical activities.

Decreased PA is associated with numerous health risks, including cardiovascular disease, type 2 diabetes, and various types of cancer (43). It is also linked to an increased risk of osteoporosis and fractures (44) as well as mental health risks such as anxiety and depression (45). To reduce the negative consequences of a pandemic or natural disasters, such as social exclusion or the closure of sports facilities that restrict PA, it is important to maintain or even increase PA levels despite the restrictions of social distancing measures. To this end, PA programs should be provided according to regional and group characteristics, even in a pandemic situation. The CHS source data used in this study was in the form of a self-report from the early 2020s of COVID-19, making it difficult to estimate a causal relationship between declines in PA during the entire pandemic and to determine the objective level of PA. However, this study can be used to create a program that can increase PA participation, even in a pandemic situation, by considering the residential area, living standards, and age groups that affect PA reduction.

PA recommendations made early in COVID-19 are not pandemic-specific. Most of the recommendations are general, established before the pandemic, and future research will need to include specific recommendations that take into account the measures each country has taken and the factors that decrease PA due to the COVID-19 pandemic.

## 5. Conclusion

This study used the Community Health Survey (CHS) raw data to investigate the association between self-reported risk factors for reducing PA during the COVID-19 pandemic. Results showed that PA decreased in all age groups, and genders, and was lower in other age groups than those in their 60s or older. Stress experience, residence area, housing type, drinking, and smoking, education level, and fear of infection were found to have an effect on decreased PA due to COVID-19. It was concluded that a PA program should be prepared considering the area of residence, economic level, life pattern, and place so that it can be used even in a pandemic situation to maintain or even increase PA.

## Data availability statement

Publicly available datasets were analyzed in this study. This data can be found at: https://chs.kdca.go.kr/chs/index.do.

## Ethics statement

Ethical review and approval was not required for the study on human participants in accordance with the local legislation and institutional requirements. The patients/participants provided their written informed consent to participate in this study.

## Author contributions

J-YS was in charge of research planning, data cleaning, data analysis, writing results, and submission. NY contributed to the composition and writing of research contents. JL contributed to the creation of figures, tables, and review of related articles. J-ML contributed to the research planning, data variable recoding, and final study conform. All authors contributed to the article and approved the submitted version.

## Conflict of interest

The authors declare that the research was conducted in the absence of any commercial or financial relationships that could be construed as a potential conflict of interest.

## Publisher’s note

All claims expressed in this article are solely those of the authors and do not necessarily represent those of their affiliated organizations, or those of the publisher, the editors and the reviewers. Any product that may be evaluated in this article, or claim that may be made by its manufacturer, is not guaranteed or endorsed by the publisher.
